# Nonbacterial Thrombotic Endocarditis (Libman-Sacks) as a Complication of Metastatic Pancreatic Cancer: A Case Report of Diagnostic and Therapeutic Challenges

**DOI:** 10.7759/cureus.96625

**Published:** 2025-11-11

**Authors:** Waleed Sultan, Mohammed Shibli, Velam Madhu, Michaeleen Wilson, Jason Burmeister

**Affiliations:** 1 Family Medicine, Conemaugh Memorial Medical Center, Johnstown, USA

**Keywords:** cancer-associated thrombosis, hypercoagulable state, : libman-sacks endocarditis, nonbacterial thrombotic endocarditis (nbte), pancreatic adenocarcinoma

## Abstract

Libman-Sacks endocarditis (LSE), or nonbacterial thrombotic endocarditis (NBTE), is a rare condition most commonly associated with systemic lupus erythematosus (SLE), but it may also occur in patients with malignancies. NBTE is an underrecognized cause of cancer-associated embolic strokes of undetermined source. We report the case of a 68-year-old female with metastatic pancreatic adenocarcinoma who presented with acute visual disturbances and headaches. Neuroimaging revealed multiple embolic infarcts in different vascular territories. Transesophageal echocardiography (TEE) identified a mass on the posterior mitral valve, consistent with sterile thrombus formation. Laboratory and imaging studies confirmed advanced pancreatic cancer, creating a hypercoagulable state likely responsible for NBTE. The patient was managed with anticoagulation using low-molecular-weight heparin and palliative chemotherapy targeting the underlying malignancy. Despite these interventions, her clinical course progressed with recurrent embolic events and systemic decline, ultimately necessitating hospice care.

This case highlights the diagnostic challenges of NBTE in malignancy, emphasizing the need to consider this condition in patients with multiple embolic strokes of varying ages. Early recognition through neuroimaging and echocardiography is essential for timely anticoagulation and multidisciplinary management. Clinicians should maintain a high index of suspicion for NBTE in patients with prothrombotic cancers, such as pancreatic adenocarcinoma, as prompt diagnosis can guide appropriate therapy and optimize quality of life.

## Introduction

Libman-Sacks endocarditis (LSE), also known as nonbacterial thrombotic endocarditis (NBTE), is a form of sterile endocarditis characterized by fibrin-platelet vegetations, most commonly affecting the mitral and aortic valves [1]. It is classically associated with autoimmune diseases such as systemic lupus erythematosus (SLE) and antiphospholipid syndrome [2]. However, LSE can also develop in the setting of malignancy, particularly in cancers with a strong prothrombotic profile, such as pancreatic adenocarcinoma [3,4]. The pathogenesis in cancer patients is thought to be driven by tumor-derived cytokines and procoagulant factors that activate the coagulation cascade and promote thrombus formation on cardiac valves [3,5,6].

Pancreatic cancer, in particular, is associated with a high risk of thromboembolic events due to its potent activation of systemic coagulation pathways [5]. These sterile vegetations may embolize, leading to serious complications such as ischemic strokes and organ infarctions. Diagnosing LSE is often difficult due to its nonspecific presentation and overlap with other causes of embolic disease. In cases with negative blood cultures and systemic embolic phenomena, transesophageal echocardiography (TEE) is the preferred imaging modality for detection [7,8].

In this report, we present the case of a patient with metastatic pancreatic adenocarcinoma who developed multiple embolic cerebrovascular accidents (CVAs) secondary to LSE. This case highlights the need for high clinical suspicion, the role of comprehensive imaging, and the importance of multidisciplinary care in managing malignancy-associated thromboembolic complications.

## Case presentation

**Figure 1:**
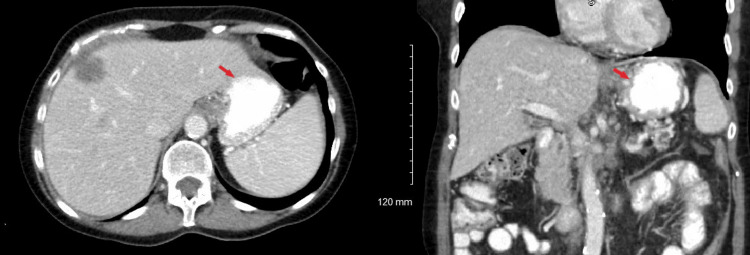
Contrast-enhanced CT abdomen and pelvis These images demonstrate a possible mass in the body of the pancreas, with a dilated distal pancreatic duct. The findings are suspicious for malignancy. Thrombosis of the splenic vein, with collateral formation, is also noted (arrows). CT: computed tomography

A 68-year-old female with a past medical history of hyperlipidemia and gastroesophageal reflux disease (GERD) and a notable family history of multiple first-degree relatives with thyroid, lung, breast, colon, prostate, and brain cancers presented for evaluation of epigastric and left lower quadrant abdominal pain. The symptoms were associated with constipation, reduced oral intake, and unintentional weight loss of approximately 10 pounds. Physical examination revealed generalized abdominal tenderness with normal bowel sounds, without rebound tenderness, palpable masses, or organomegaly. Computed tomography (CT) of the abdomen and pelvis with intravenous contrast demonstrated a pancreatic mass with associated hepatic metastases, thrombosis of the splenic vein, and omental caking (Figure 1). Laboratory testing revealed an elevated cancer antigen (CA) 19-9 level of 715 U/mL, CA-125 of 302 U/mL, and CA 27.29 of 239.6 U/mL, while carcinoembryonic antigen (CEA) was within normal limits (Table 1).

**Table 1:** Laboratory results of outpatient workup WBC: white blood cell count; CA: cancer antigen; CEA: carcinoembryonic antigen

Test	Result	Normal Range	Interpretation
WBC	5.98	4.5 - 11.0 x 10³/μL	Normal
Hemoglobin	12.3	12 - 16 g/dL	Normal
Platelets	184	150 - 450 x 10³/μL	Normal
Sodium	136	135 - 145 mEq/L	Normal
Potassium	4.1	3.5 - 5.0 mEq/L	Normal
Creatinine	0.60	0.6 - 1.2 mg/dL	Normal
Lipase	29	12 - 53 U/L	Normal
CA 19-9	715	0 - 35 U/mL	Elevated (suggestive of malignancy)
CA 125	302	0 - 38.1 U/mL	Elevated (suggestive of malignancy)
CA 27.29	239.6 U/mL	0 - 38.6 U/mL	Elevated
CEA	< 2	0 - 5	Normal

**Figure 2:**
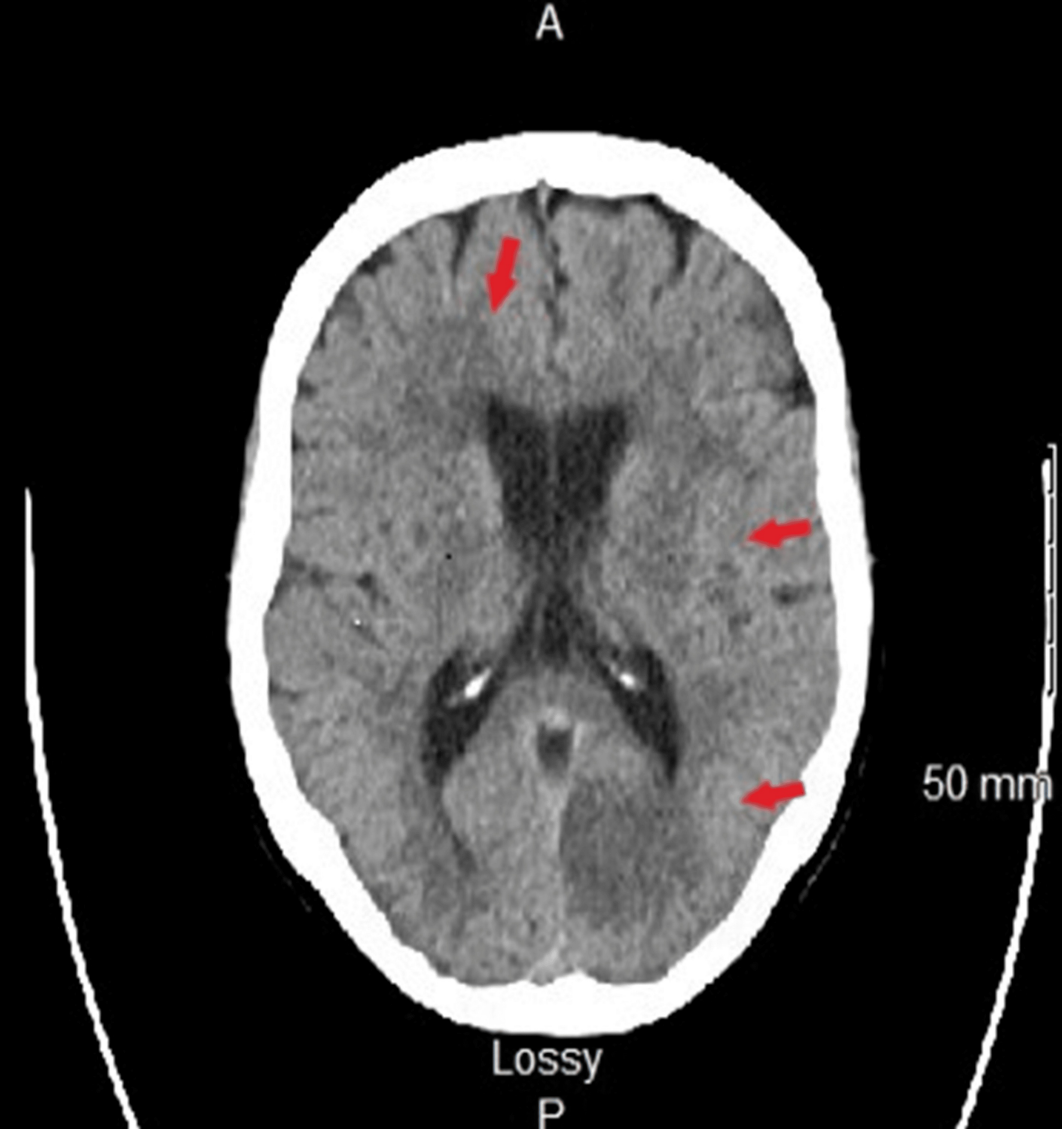
Axial non-contrast CT of the head This image shows patchy hypodensities in the right frontal and left parietal lobes (arrows), consistent with acute infarcts. CT: computed tomography

The patient subsequently developed sudden-onset visual disturbances upon awakening, characterized by shading and loss of vision in the right temporal visual field. An outpatient ophthalmologic evaluation raised concerns about a neurological etiology, prompting a referral to the Emergency Department (ED) for further assessment. In the ED, non-contrast CT of the head revealed patchy hypodensities in the right frontal and left parietal lobes, concerning for acute infarcts (Figure 2). The patient was admitted to the General Medicine Department for further evaluation and management. On admission, physical examination findings and laboratory results are summarized in Tables 2-3, respectively.

**Table 2:** Physical exam findings on admission

System	Findings
Constitutional	Underweight, not ill-appearing
Eyes	Shading/vision loss in the right temporal visual field
Cardiovascular	Regular heart rhythm, no murmurs, normal pulses
Pulmonary	Normal breath sounds, no wheezing or rhonchi
Abdominal	No distension, no palpable mass, abdominal tenderness
Neurological	Visual field deficits, no focal deficits
Skin	Pale
Psychiatric	Behavior normal

**Table 3:** Laboratory results on admission WBC: white blood cell count; CK: creatine kinase; PT: prothrombin time; INR: international normalized ratio

Test	Results	Normal Range	Interpretation
WBC	8.90	4.5 - 11.0 x 10³/μL	Elevated
Hemoglobin	13.4	12 - 16 g/dL	Normal
Platelets	199	150 - 450 x 10³/μL	Normal
Sodium	132	135 - 145 mEq/L	Low
Creatinine	0.70	0.6 - 1.2 mg/dL	Normal
CK	34	32 - 190 U/L	Normal
PT	11.9	9 - 12 sec	Normal
INR	1.1	0.8 - 1.1	Normal
Blood cultures × 2	Negative throughout the admission		
Troponin	369	0 - 34 ng/L	Elevated
Urinalysis	Unremarkable		
Hepatic function panel	Within normal limits		

**Figure 3:**
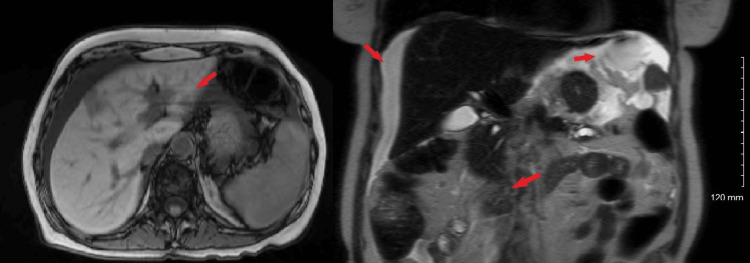
MRI of the abdomen with and without contrast These images demonstrate an ill-defined malignant neoplasm in the body of the pancreas, with distal pancreatic ductal dilatation (arrows). Additional findings include hepatic and omental metastases, mild ascites, abdominal lymphadenopathy, and left adrenal gland thickening, suggestive of metastatic disease. MRI: magnetic resonance imaging

Further diagnostic workup included magnetic resonance imaging (MRI) of the abdomen, with and without contrast, which demonstrated an ill-defined malignant neoplasm in the body of the pancreas, with associated distal pancreatic ductal dilatation. Additional findings included hepatic and omental metastases, mild ascites, abdominal lymphadenopathy concerning for metastatic disease, and left adrenal gland thickening, also suggestive of metastatic involvement (Figure 3). Ultrasound-guided fine-needle aspiration of a hepatic lesion confirmed adenocarcinoma involving the liver tissue, favoring a pancreaticobiliary primary origin.

**Figure 4:**
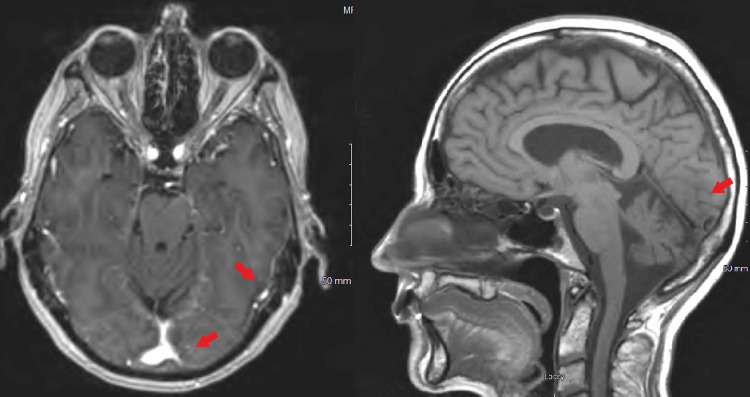
MRI of the brain with and without contrast These images demonstrate an acute infarct in the left occipital lobe (arrows). Additional small punctate areas of acute infarction are present in the left parietal deep white matter and left thalamus, which are findings suggestive of an embolic phenomenon. A small enhancing focus is also noted in the right parietal region, which may represent a prominent dural vessel; however, a small focus of dural metastasis cannot be excluded. MRI: magnetic resonance imaging

MRI of the brain, with and without contrast, revealed a subacute infarct in the left occipital lobe (Figure 4). Collectively, these findings established the diagnosis of metastatic pancreatic adenocarcinoma with associated embolic cerebrovascular events.

**Figure 5:**
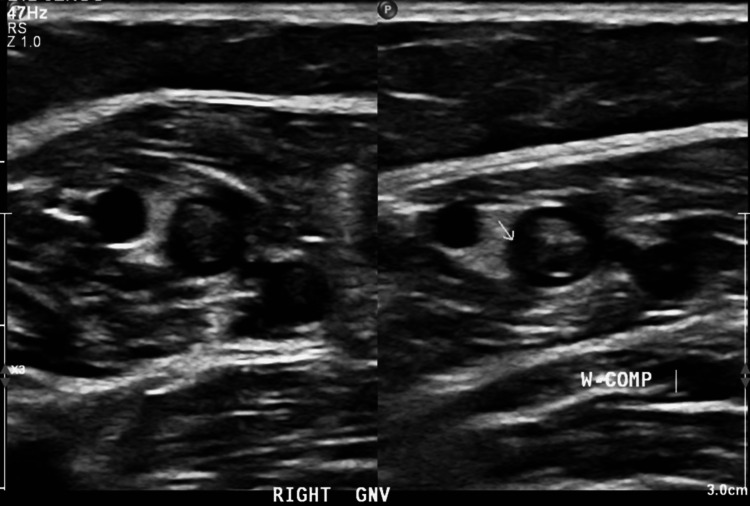
Duplex venous ultrasound of the right lower extremity This image demonstrates acute deep vein thrombosis within the gastrocnemius and soleal veins.

**Figure 6:**
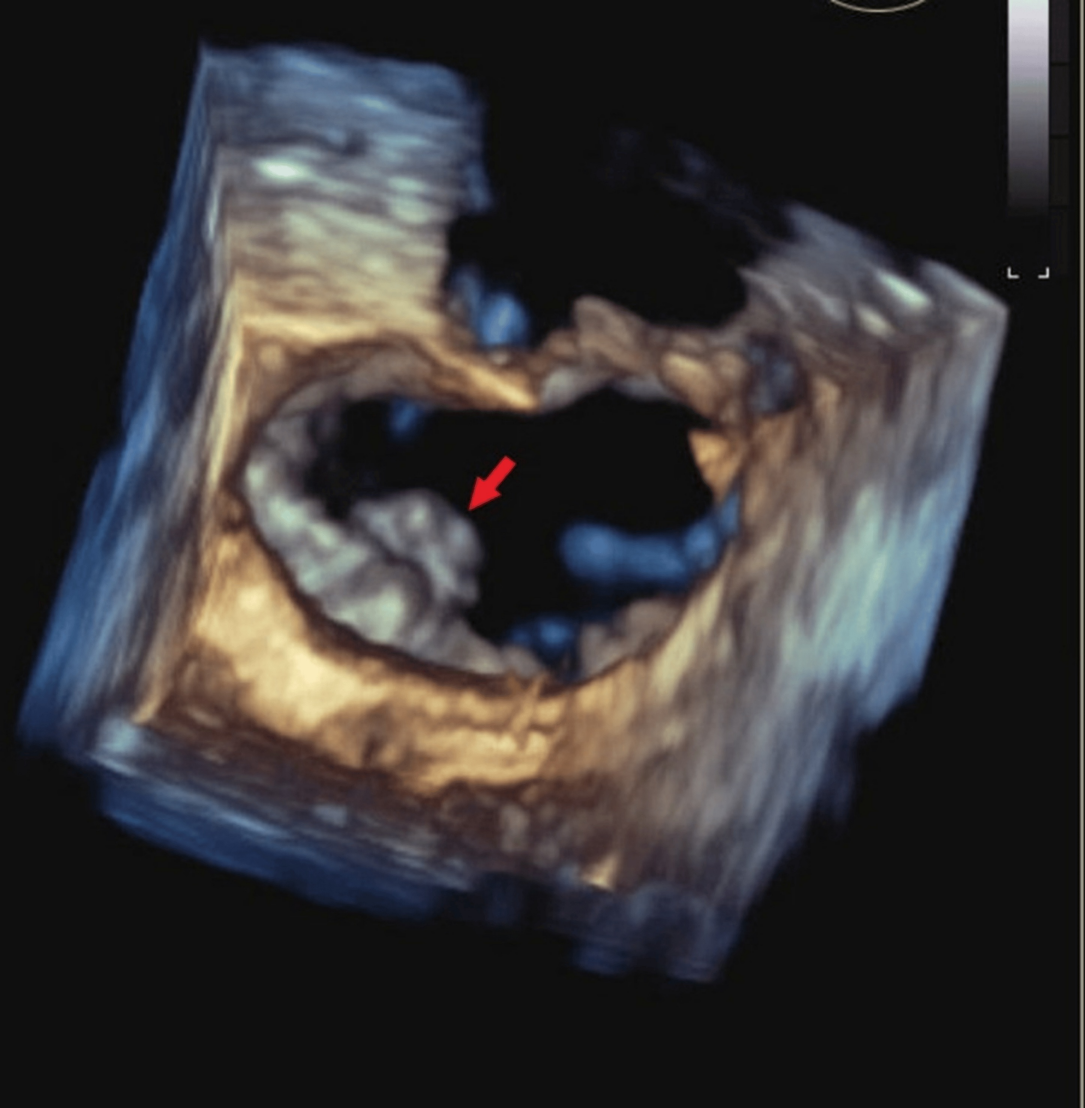
Transesophageal echocardiography This image demonstrates a mass on the atrial side of the posterior mitral valve leaflet (P2), measuring 8 × 5 mm (arrow) and attached to the base of the leaflet. No significant mitral regurgitation is associated with this finding.

A comprehensive stroke workup was conducted. A transthoracic echocardiogram (TTE) with bubble study was unremarkable. Venous duplex ultrasound of the lower extremities revealed acute deep vein thrombosis (DVT) in the right gastrocnemius and soleal veins (Figure 5). TEE demonstrated a mass on the atrial side of the posterior mitral valve leaflet (P2 segment), measuring approximately 8 × 5 mm, without evidence of significant mitral regurgitation (Figure 6). Given the negative results from initial and repeat blood cultures, and the absence of clinical signs or symptoms of infection, the mitral valve lesion was consistent with NBTE, a condition frequently associated with malignancies, particularly pancreatic cancer.

During the hospital course, the patient was initiated on anticoagulation therapy with low-molecular-weight heparin for treatment of the diagnosed DVT and NBTE. In light of the advanced stage of her pancreatic cancer, the oncology team recommended palliative chemotherapy, and the patient received her first cycle. Following treatment, she developed worsening nausea, which was managed with antiemetic therapy. Despite medical management, the patient’s clinical status continued to decline, and she became progressively cachectic. Given the progression of her disease and poor overall prognosis, a decision was made to transition her to hospice care for comfort-focused management.

At discharge, the patient was stable but continued to experience symptom progression. She was provided with comfort measures, including a Foley catheter, morphine, lorazepam, and medications for nausea and gastrointestinal symptoms. Hospice care was arranged for ongoing symptom management at home, with family involvement in care planning.

## Discussion

LSE, or NBTE, is most commonly associated with autoimmune diseases such as SLE and antiphospholipid syndrome [1,2]. However, LSE can also develop in patients with malignancies, particularly in cancers with a strong prothrombotic profile, such as pancreatic adenocarcinoma [3,4,6]. Tumor-associated hypercoagulable factors contribute to the formation of sterile thrombi on heart valves, which may embolize to distant organs, resulting in ischemic events [3,5]. This case illustrates the diagnostic challenges and clinical management of LSE in a patient with metastatic pancreatic adenocarcinoma.

Pathophysiology and mechanism in cancer patients

Pancreatic cancer is a well-established prothrombotic condition that can precipitate thrombotic events, including NBTE [3,4]. Tumor-derived procoagulant factors, including cytokines and mucins, activate the coagulation cascade, promoting the formation of sterile vegetations on cardiac valves [5]. In this patient, metastatic pancreatic adenocarcinoma likely contributed to multiple embolic CVAs via thrombus formation on the posterior mitral valve, resulting in ischemic brain damage [3,4,7].

Diagnostic challenges

Diagnosing LSE in patients with malignancy is challenging due to nonspecific presentations and overlap with other embolic conditions [3]. The differential diagnosis includes infective endocarditis (IE), papillary fibroelastoma (PFE), and Lambl’s excrescences [1]. IE was excluded by negative serial blood cultures, the absence of fever or inflammatory markers, and echocardiographic findings inconsistent with infection. PFE and Lambl’s excrescences were unlikely, given their smaller, pedunculated morphology and lower embolic risk. Our patient presented with neurological deficits, including headaches and visual disturbances, which prompted neuroimaging. CT imaging revealed multiple embolic infarcts (Figure 1), including a subacute infarct in the left occipital lobe and scattered subcortical infarctions, suggestive of embolic phenomena. TEE confirmed a mass on the posterior mitral valve (Figure 2), consistent with NBTE. Echocardiography remains the standard diagnostic modality for LSE, even in patients with malignancy [5,6,8].

Management

Management of LSE in cancer patients requires a multidisciplinary approach, focusing on anticoagulation and treatment of the underlying malignancy [2,3]. In this patient, anticoagulation with low-molecular-weight heparin (LMWH, Lovenox) was initiated to prevent further embolic events. In malignancy-associated NBTE, LMWH remains the preferred anticoagulant due to its superior efficacy in preventing recurrent embolic events and its additional antitumor-related antithrombotic properties [2,3,6]. Although direct oral anticoagulants (DOACs) and antiplatelet agents have been trialed in cancer-associated thrombosis, evidence indicates limited benefit in NBTE, with reports of recurrent emboli despite therapeutic dosing [7,8]. Therefore, LMWH was selected in this case as the most evidence-based and clinically appropriate option.

Palliative chemotherapy was started to address metastatic pancreatic cancer and potentially reduce thrombotic risk. Despite these interventions, the patient's condition progressed, and she ultimately required hospice care due to the advanced disease [4,7].

This case underscores the importance of recognizing the hypercoagulable state in pancreatic cancer patients, which predisposes them to thromboembolic complications, such as NBTE [3-5,7]. Early recognition, comprehensive imaging, and multidisciplinary management involving oncology, cardiology, and neurology are essential to mitigate complications and optimize patient outcomes [2,3,5,7,9].

## Conclusions

LSE is a rare but clinically significant cause of embolic strokes, particularly in patients with underlying malignancies, such as pancreatic adenocarcinoma. Early recognition and prompt management are essential; however, prognosis remains poor in the setting of advanced cancer. This case highlights the diagnostic complexity and therapeutic challenges of LSE in oncology patients and reinforces the need for heightened clinical vigilance and multidisciplinary management. More case reports are needed to enhance clinical understanding, guide early detection strategies, and inform evidence-based management. Future research should focus on identifying reliable biomarkers and developing screening protocols for thromboembolic complications in high-risk cancer populations to facilitate earlier diagnosis and improve clinical outcomes. 
